# Echocardiographic characteristics and evidence of ventricular remodeling in subclinical insulin-dysregulated Warmblood horses

**DOI:** 10.1093/jvimsj/aalag229

**Published:** 2026-10-10

**Authors:** Megan G Palmisano, Claire Underwood, Jeaneen Sage, Darko Stefanovski, Andrew van Eps, JoAnn Slack

**Affiliations:** Department of Clinical Studies, New Bolton Center, University of Pennsylvania School of Veterinary Medicine, Kennett Square, PA 19348, United States; Department of Molecular Biomedical Sciences, College of Veterinary Medicine, North Carolina State University, Raleigh, NC 27607, United States; Department of Clinical Studies, New Bolton Center, University of Pennsylvania School of Veterinary Medicine, Kennett Square, PA 19348, United States; Department of Clinical Studies, New Bolton Center, University of Pennsylvania School of Veterinary Medicine, Kennett Square, PA 19348, United States; Department of Clinical Studies, New Bolton Center, University of Pennsylvania School of Veterinary Medicine, Kennett Square, PA 19348, United States; Department of Clinical Studies, New Bolton Center, University of Pennsylvania School of Veterinary Medicine, Kennett Square, PA 19348, United States; Department of Clinical Studies, New Bolton Center, University of Pennsylvania School of Veterinary Medicine, Kennett Square, PA 19348, United States

**Keywords:** cardiac hypertrophy, equine metabolic syndrome, laminitis, left ventricle

## Abstract

**Background:**

In humans, systemic hypertension and left ventricular (LV) hypertrophy are common sequelae of metabolic syndrome and pre-diabetes warranting investigation in horses with insulin dysregulation (ID).

**Hypothesis/Objectives:**

To compare echocardiographic variables, non-invasive blood pressure (NIBP), and adipokines between Warmblood (WB) horses with and without ID.

**Animals:**

Nineteen client-owned WB horses, 5-15 years of age, presenting for elective procedures were included. Horses had no history of metabolic disease, ID, laminitis, renal or cardiac disease.

**Methods:**

Blinded cross-sectional study. Horses were divided into 2 groups ID and non-ID based on an oral sugar test (T0 insulin ≥20 μIU/mL or T60 or T90 ≥ 30 μIU/mL). Each horse underwent the following: Physical examination, body condition score, body weight, cresty neck score, and measurement of serum leptin, total, and high molecular weight adiponectin concentrations, NIBP, lateromedial radiographs, retroperitoneal fat measurement, short axis M mode echocardiographic evaluation, and 2D speckle tracking (2DST).

**Results:**

Eight horses were categorized as ID, 11 as non-ID. Evidence of LV remodeling was identified in ID horses (*P* < .05) with greater measurements of LV internal diameter at end diastole (ID 11.77 cm [11.2-12.3]; non-ID 11.10 [10.54-11.85]), interventricular septum at end diastole (ID 3.10 cm [2.81-3.39]; non-ID 2.46 [2.22-2.71]) and end systole (ID 5.18 cm [4.81-5.56]; non-ID 4.29 [4.06-4.51]). 2DST measures were significantly lower in the ID group (*P* < 0.05). Hypertension was identified in 2 horses, 1 from each group.

**Conclusions and clinical importance:**

Horses with mild ID have evidence of LV remodeling supportive of hypertrophy. Cardiovascular changes might be included in the overarching definition of ID.

## Introduction

Equine metabolic syndrome (EMS) is defined as a collection of factors that predispose horses to the development of laminitis.^1^ This syndrome encompasses insulin dysregulation (ID), obesity, regional adiposity, and abnormal plasma lipid concentrations.^1–3^ ID is understood to be multifactorial with both genotypic and environmental contributions to the disease.^1^^,^^4–7^ In horses the metabolic syndrome phenotype includes adiposity, ID characterized by both hyperinsulinemia and insulin resistance, adipokine dysregulation (adiponectin and leptin), abnormal/high concentrations of inflammatory cytokines, and hypertension.^1^^,^^5^ Tachycardia and cardiomyopathy are rarely reported in horses and most often described as a consequence of hypertension secondary to laminitis or chronic kidney disease and not as a primary disease process or sequelae of ID.^8^ Heart rate (HR) elevation has similarly been attributed to pain secondary to laminitis in both horses and ponies with ID.^3^^,^^9–12^ Whether changes in HR occur secondary to ID disease pathophysiology independent of laminitis is unknown. Humans with metabolic syndrome, ID, and pre-diabetes exhibit a similar phenotype to affected horses with metabolic syndrome or ID.^13–17^ Based on what is established in humans with metabolic syndrome and pre-diabetes, systemic hypertension and left ventricular (LV) hypertrophy are common sequelae warranting a similar investigation of horses with ID.^18–21^

In ponies LV hypertrophy, as supported by changes to relative wall thickness (RWT) and mean wall thickness (MWT), are hypothesized to be associated with both presence of laminitis and resulting hypertension in EMS and ID.^12^^,^^22^ It is unknown whether these associations are specific to ponies, or if they are also present in horses of other breeds affected by ID. A pilot study in horses with ID identified changes to myocardial function based on tissue Doppler interrogation, suggestive of probable effects of ID on the heart in full size horses.^23^ In comparison to other breeds, there is predilection for ID in Warmblood (WB) horses,^6^^,^^24–26^ therefore preliminary investigations of associations between ID and cardiovascular dysfunction are warranted in this group.

The primary objective of this study was to compare echocardiographic variables and non-invasive blood pressure (NIBP) between WB horses with and without ID. We hypothesized that horses with ID would have greater measurable echocardiographic variables associated with cardiac hypertrophy and measurable hypertension than horses without ID, even in the absence of laminitis. Additional objectives include first to examine for associations between NIBP and echocardiographic variables, second to evaluate possible associations between cardiac changes and markers of metabolic health (serum adiponectin and leptin, and ultrasonographic fat measurements) aside from insulin, and third to determine whether there are associations between a diagnosis of laminitis and underlying cardiac changes in WB horses. The goal of the study was to provide further insight into the implications of mild or undiagnosed ID on equine cardiac function and to determine whether currently available hematologic biomarkers are associated with changes in echocardiographic variables.

## Materials and methods

### Animals

A blinded cross-sectional study design was utilized for this investigative study. Client-owned WB horses 5-15 years of age presenting to a tertiary referral center for elective procedures were included. The study was granted ethical approval by the University of Pennsylvania’s Institutional Animal Care and Use Committee (IACUC #807119).

### Experimental design

The following variables were each performed: physical examination, body condition score (BCS 0-9), body weight, cresty neck score (CNS, 0-5),^27^^,^^28^ oral sugar test (OST) to define horses as either ID or non-ID, leptin, adiponectin, high molecular weight (HMW) adiponectin, non-invasive oscillometric blood pressure measurements, lateromedial foot radiographs of both front feet, retroperitoneal fat measurements, abdominal fat measurements, and echocardiographic M-mode views. 2D speckle tracking (2DST) was performed in 7 horses from each group.

### Echocardiographic measurements

All echocardiographic measurements were performed in triplicate on non-consecutive cardiac cycles and averaged. M-mode views were obtained from right parasternal short axis of the left ventricle (LV) at the level of the chordal attachments. Measurements of the LV chamber diameter, LV free wall thickness (LVFW) and interventricular septal thickness (IVS) were performed in both systole (s) and diastole (d). Fractional shortening (FS), mean wall thickness (MWT = LVFWd+IVSd), left ventricular mass (LVM = 1.04^*^ (((LVIDd+LVFWd+ IVSd)^3^-(LVIDd^3^))-13.6)), and relative left ventricular wall thickness (RWT = ((LVFWd+IVSd)/LVIDd)) were calculated based on the previously reported formulas.^29^^,^^30^ Pulsed wave tissue Doppler imaging (PW-TDI) was used to obtain LV myocardial velocities and cardiac cycle intervals using the right parasternal short axis view of the LV at the level of the chordae tendineae as previously reported.^31^ The sample volume was placed on the left ventricular free wall. Diastolic function was assessed based on measurements of IVRTm (isovolumic relaxation time), Em (early-diastolic LV wall motion velocity during the phase of rapid ventricular filling), and Am (late-diastolic LV wall motion velocity at the time of atrial contraction). Em/Am was calculated.

### Statistical analysis

All analyses were conducted with Stata 16.1MP (StataCorp) with *P*-value < .05 as the criterion for statistical significance. Inference statistical analysis was based on a multiple linear regression model where the fixed effect was the presence or absence of ID defined as a categorical variable with age (continuous variable) and presence or absence of laminitis as confounders. Results were reported as marginal (model adjusted) means with their respective 95% CI.

Detailed experimental methodology is provided in Appendix A.

## Results

A total of 19 client-owned horses were enrolled in the study. Horses included in the study presented for elective neurologic evaluation (*n* = 8), poor performance and advanced diagnostic imaging (*n* = 8), ophthalmic evaluation (*n* = 1), gastroscopy (*n* = 1), and surgical consult for ethmoid hematoma (*n* = 1). None of the horses were in work at the time of presentation. No horses were maintained on non-steroidal anti-inflammatories before presentation. Medications administered before evaluation included omeprazole (*n* = 2), and vitamin E (Elevate, *n* = 2). All horses had normal cardiac auscultation at time of examination with no murmurs or arrhythmias.

Horses were divided into 2 groups based on the results of the OST. A total of 8 WB horses were categorized as ID with 11 as non-ID. The ID group included 4 mares and 4 geldings, with a median age of 10.5 years (range 6-15). The ID group mean insulin concentrations were 19.9 with 95% CI [12.1-27.6] μIU/mL at T0, 48.2 μIU/mL [31.7-64.7] at T60, and 41.5 μIU/mL [26.2-56.8] at T90. The non-ID group included 11 WB horses, 7 mares, and 4 gelding, with a median age of 6 years (range 5-11). The non-ID horse insulin concentrations were 7.0 μIU/mL [3.1-10.9] at T0, 13.8 μIU/mL [9.0-18.5] at T60, 13.2 μIU/mL [8.6-17.9] at T90 (Figure 1). Descriptive statistics for cardiovascular variables and results of statistical analysis are listed in Table 1.

**Figure 1:**
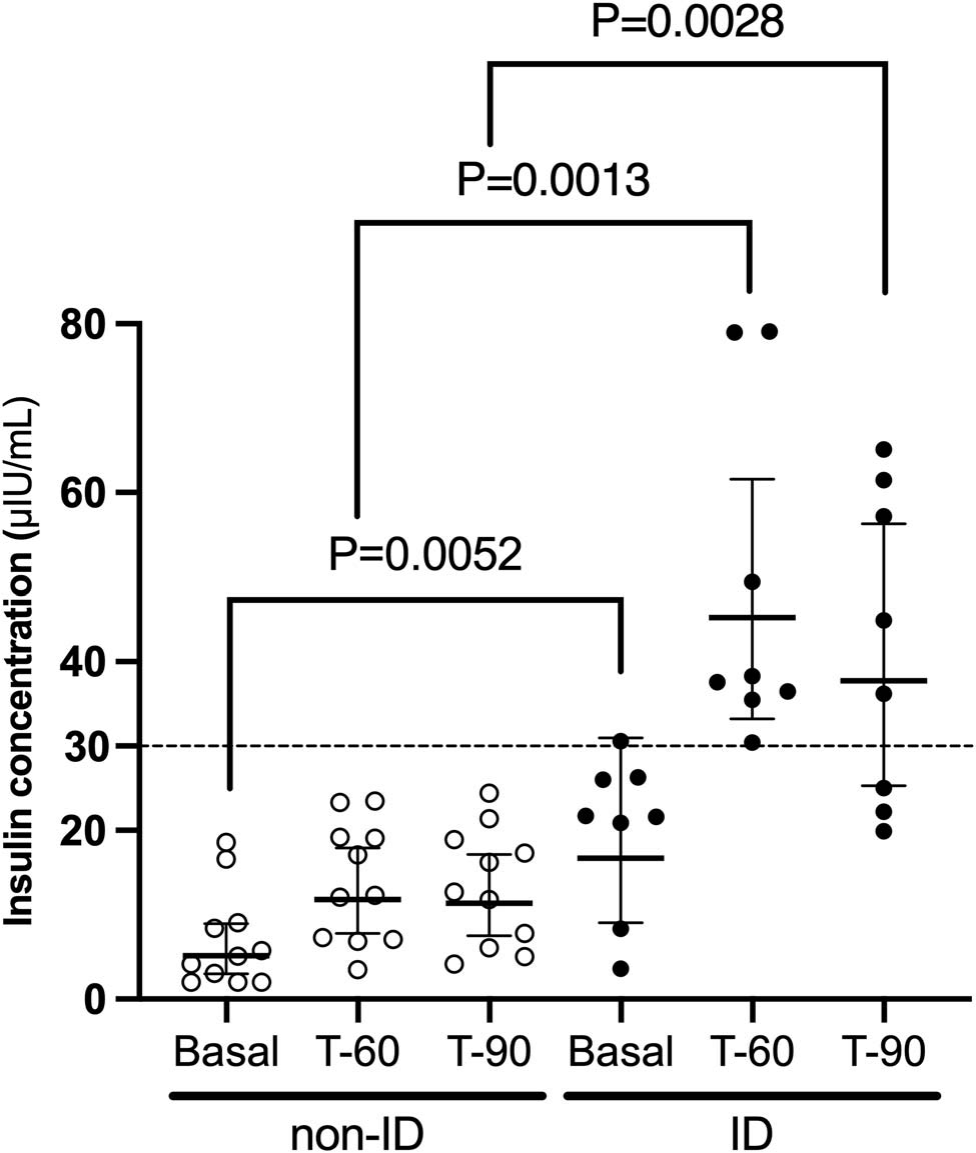
Oral sugar test insulin concentrations. Insulin concentrations of all enrolled WB horses during oral sugar test (OST). Basal, 60 and 90 min after karo syrup administration insulin concentrations are reported for both non-ID and ID horses in μlU/mL. Horizontal bars represent the marginal means and 95% CIs. *∘ * Non-ID. • ID.

**Table 1:** Echocardiographic variables in ID and non-ID horses.

Variable	ID Mean [95% CI]	Non-ID Mean [95% CI]	*P*-value
**Mean heart rate during echo (bpm)**	47.5 [42.4-56.2]	40.3 [39-48.3]	.068
**LVIDd (cm)**	11.77 [11.2-12.3]	11.10 [10.54-11.85]	**.038**
**IVSs (cm)**	5.18 [4.81-5.56]	4.29 [4.06-4.51]	**<.001**
**IVSd (cm)**	3.10 [2.81-3.39]	2.46 [2.22-2.71]	**.007**
**LVFWs (cm)**	4.74 [4.08-5.41]	4.08 [3.58-4.57]	**.025**
**LVFWd (cm)**	2.6 [2.33-2.88]	2.38 [2.15-2.61]	.1
**FS (%)**	45.78 [42.4-49.13]	42.8 [40.49-45.1]	.05
**MWT (cm)**	2.85 [2.61-3.09]	2.4 [2.21-2.64]	**.014**
**LVM (g)**	3848.66 [3387.56-4309.75]	2897.96 [2340.03-3455.88]	**.005**
**RWT (cm)**	0.48 [0.43-0.54]	0.43 [0.4-0.46]	.16
**E:A ratio**	0.58 [0.28-0.88]	0.64 [0.43-0.85]	.16
**Radial strain average (%)**	49.39 [41.07-57.71]	66.92 [61.01-72.84]	**.024**

ID and non-ID values are represented in each bolded column groups. *P*-values are calculated using ID and non-ID as fixed effects, with those bolded identified as significant (<.05). Means are reported as the marginal means.

Statistical significance was established across multiple cardiovascular variables between ID and non-ID horses including LVIDd, IVSs, IVSd, LVFWs, MWT, LVM, and peak systolic radial strain (Table 1). When allometric scaling was performed (Table 2) to assess for diameter associated cardiovascular variables (LVIDd, IVSs, IVSd, LVFWs, LVFWd, MWT, LVM) significant differences were detected for LVIDd and IVSs (Table 2).

**Table 2:** Allometric scaling of echocardiographic variables in ID and non-ID horses.

Variable	ID Mean [95% CI]	Non-ID Mean [95% CI]	*P*-value
**LVIDd (cm)**	11.05 [10.38-11.72]	10.54 [9.85-11.24]	.073
**IVSs (cm)**	4.79 [4.56-5.02]	4.18 [3.98-4.38]	**<.001**
**IVSd (cm)**	2.80 [2.56-3.04]	2.44 [2.21-2.66]	**.021**
**LVFWs (cm)**	4.41 [4.01-4.81]	4.12 [3.78-4.46]	.084
**LVFWd (cm)**	2.56 [2.10-3.01]	2.36 [2.17-2.55]	.361
**MWT (cm)**	2.68 [2.41-2.95]	2.40 [2.20-2.59]	.068
**LVM (g)**	3193 [2721-3666]	2576 [2100-3053]	.05

ID and non-ID values are represented in each bolded column groups. *P*-values are calculated using ID and non-ID as fixed effects, with those bolded identified as significant (<.05). Means are reported as the marginal means.

Two horses, both meeting criteria for ID, had laminitis at time of evaluation without a history of known laminitic changes (Table 1A). The horses were 12 and 13 years of age. These horses NIBP values were within normal reference range with mean arterial pressure (MAP) of 92.7 mmHg and 106.2 mmHg, systolic pressure of 117 mmHg and 139.8 mmHg, and diastolic pressure of 79.3 mmHg and 87.17 mmHg respectively. One of the horses was over conditioned with a BCS of 7/9, and the greatest intraabdominal fat measurement of 4.8 cm. Both horses had low HMW adiponectin concentrations of 4.56 and 5.48 mg/L. Echocardiographic measurements for both horses were included within the 95% CI for the ID population (Table 1).

Height corrected NIBP is represented in Figure 2. No statistical significance was established when comparing systolic, diastolic, and mean blood pressure (BP) between ID and non-ID groups. Based on studies in horses,^2^^,^^8^^,^^32–34^ 1 horse each from the ID and non-ID groups was considered hypertensive at the time of measurement.

**Figure 2:**
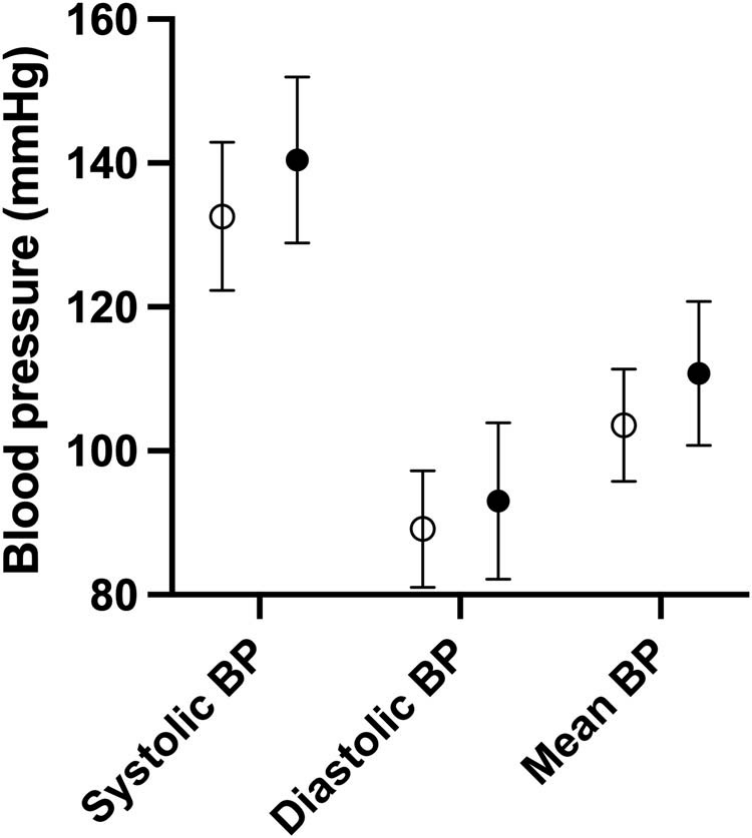
Height corrected non-invasive blood pressure. Height corrected non-invasive blood pressure (BP) measurements for insulin dysregulate (ID) and non-ID horses. Closed and open circles represent marginal means measurements, with the upper and lower bars representative of the 95% confidence interval. Measurements for systolic, diastolic and mean arterial pressure (mmHg) for the non- ID group were 132.6 [122.33-142.87], 89.14 [81.03-97.26], and 103.56 [95.76-111.34], respectively, and 140.4 [128.89-151.96], 93 [82.18-103.89], and 110.79[100.79-120.78], respectively in the non-ID group. *∘ * Non-ID. • ID.

Metabolic variables are represented in Table 3. CNS was significantly different between ID and non-ID groups (*P* = .005). No statistical significance was found when investigating other metabolic variables between ID and non-ID groups. Using 2DST analysis of the LV in short-axis, significant differences were detected for Lat, Post, and Inf (Table 4).

**Table 3:** Left ventricle 2D speckle tracking (2DST) Q-analysis.

Variable	ID Mean [95% CI]	Non-ID Mean [95% CI]	*P*-value
**AntSept**	54.23 [50.2-66.53]	67.81 [62.67-72.64]	.053
**Ant**	50.14 [45.06-70.95]	63.7 [61.02-73.51]	.16
**Lat**	42.92 [37.79-52.26]	61.89 [53.46-73.57]	**.038**
**Post**	42.92 [36.08-48.10]	61.71 [52.5-69.7]	**.026**
**Inf**	43.92 [39.96-50.15]	60.42 [56.1-66.85]	**.017**
**Sept**	48.28 [44.84-61.97]	67.13 [61.19-68.18]	.053

Q-analysis of 2DST divides the right-parasternal short-axis view of the left ventricle at the level of the chordae tendineae into 6 segments as defined in the variable column. *P*-values are calculated using ID and non-ID as fixed effects, with those bolded identified as significant (<.05).

**Table 4:** Metabolic variables.

Variable	ID Mean [95% CI]	Non-ID Mean [95% CI]	*P*-value
**Abd. fat (cm)**	2.66 [1.58-3.74]	1.69 [1.07-2.31]	.099
**RP fat (cm)**	2.71 [1.87-3.54]	2.43 [1.61-3.26]	>.2
**Leptin (ng/mL)**	12.08 [3.77-20.40]	12.8 [1.59-24.02]	>.2
**Total adiponectin (mg/L)**	12.41 [9.62-15.21]	15.4 [52.5-69.7]	.191
**HMW adiponectin (mg/L)**	7.2 [2.92-11.49]	10.75 [8.19-13.32]	.056
**BCS**	6.62 [6.19-7.06]	5.73 [4.99-6.47]	.055
**CNS**	2.0 [1.23-2.77]	0.82 [0.41-1.22]	**.005**

ID and non-ID values are represented in each bolded column group. *P*-values are calculated using IF and non-ID as fixed effects, with those bolded identified as significant (<.05). Means are reported as the marginal means.

## Discussion

This echocardiographic study investigates LV echocardiographic measurements in WB horses with mild ID. The differences between both the ID and non-ID groups, although statistically significant, were not outside of previously established reference ranges.^31^^,^^35^^,^^36^ Multiple studies have identified evidence of cardiac hypertrophy in ponies and horses with ID.^3^^,^^12^^,^^23^ Based on the findings of this study there is a strong association between possible LV hypertrophy as defined by changes to multiple echocardiographic variables (eg, LVIDd, IVSd, IVSs) and ID. This finding suggests that LV hypertrophy might occur secondary to ID, although it could be due to a third systemic abnormality in these horses.

In the WB horses sampled, a diagnosis of ID was associated with changes to LV cardiac variables consistent with cardiac hypertrophy (Table 1, LVIDd, IVSd, IVSs, LVFWs, MWT, LVM, radial strain), particularly increases in IVSd and IVSs and IVSd (*P* = .007 and <.001 respectively). Horses with an IVSs above 4.9 cm was strongly correlated with a diagnosis of ID (ref 4.3 ± 0.47.^35^), although within the 90% CI previously established.^35^ When allometric scaling was performed on the same echocardiographic variables, significant differences were limited to IVSd and IVSs (*P* < .001 and .021, respectively) (Table 2). When horse size was taken into account a number of LV variables were no longer statistically significant between groups, including LVM and MWT, both of which are supportive of global LV changes.^37^^,^^38^ Allometric scaling allowed for size-independent physiological echocardiographic measures to better interpret the statistical significance obtained via echocardiographic measurements. Only one previous echocardiographic study in WB horses utilized allometric scaling to investigate obtained echocardiographic measurements,^36^ limiting the interpretation of raw echocardiographic measures compared to those obtained via allometric scaling. However, despite normalization of multiple LV variables between the ID and non-ID horses after analysis with allometric scaling, comparing the 2 methodologies further supports the changes in the IVS both in diastole and systole between the ID and non-ID horses as being noteworthy and likely clinically relevant. Previous echocardiographic studies of healthy horses do not include metabolic testing as an echocardiographic factor in their definition of healthy horses.^31^^,^^35^^,^^36^ This could be considered in future echocardiographic studies of horses given the findings of this study.

Measurements from a short axis M-mode of the LV can provide insight to early development of LV changes and hypertrophy in equids based on what is known in other species. In this study the 17% higher IVSs in ID compared to non-ID horses was the most prominent echocardiographic difference, where previous studies have focused on the LVFW during diastole as the major echocardiographic measurement to assess cardiac hypertrophy.^12^ In dogs thickened IVSd and IVSs are associated with a diagnosis of hypertrophic cardiomyopathy (HCM), with a thickened IVSd found in all postmortem examinations in dogs with a diagnosis of HCM.^39^ Horses with ID most resemble pre-diabetic human patients, although more is known about diabetic cardiomyopathy than those that are pre-diabetic. In humans with diabetic cardiomyopathy, the earliest manifestation of disease is LV hypertrophy and thickening of the interventricular septum secondary to myocardial hypertrophy and lipid accumulation within the myocardium.^40–42^ Additionally, in humans, IVSd and IVSs have greater sensitivity for detection of early LV changes and early diagnosis of LV hypertrophy, which is more relevant to the horses included in this study based on the mild ID identified.^43^^,^^44^ Horses with mild and subclinical ID could be expected to have more mild cardiac changes compared to those with severe disease or disease of longer duration. Using information available from a previous study in equids diagnosed with hypertensive cardiomyopathy RWT, MWT, LVM, and IVSd (5/5) were increased in all horses while IVSs was increased in 4 of 5 horses.^8^ This pattern suggests that IVSd might be a more sensitive echocardiographic indicator of cardiac hypertrophy in horses, consistent with observations in humans.^8^^,^^43^^,^^44^

Other potential measures of LV hypertrophy were evaluated including Em/Am ratio. Em/Am ratio was inconsistent in this study, likely due to variation in the degree of LV remodeling and hypertrophy. Em/Am ratios can be normal in humans with diagnosed ventricular hypertrophy.^45^ 2DST and radial strain measurements have been examined in horses, to investigate cardiac function in cases of aortic regurgitation, mitral regurgitation, and myocardial disease.^31^^,^^46–48^ In humans LV remodeling and subclinical disease are identified using 2DST, underscoring the usefulness of this diagnostic modality.^49^ Measurements obtained using 2DST were significantly lower in the ID group compared to the non-ID group (Table 3) suggestive of LV systolic dysfunction.

There are multiple potential explanations for the significant differences between the ID and non-ID horses including difference in horse age, which were significantly different in the ID and non-ID groups (*P* = .01). However horses in this study were less than 15 years of age compared to other echocardiographic studies where age ranged from 3 to 25^35^ or 6-23^36^ with median ages of 8 and 12, respectively. Previous echocardiographic studies^31^^,^^36^ establishing reference intervals in WB horses do not take into consideration the presence of ID, or other metabolic disturbances, when determining exclusion criteria. Based on the large age ranges used to establish normal echocardiographic variables in WB, there is a chance that a portion of horses used for establishing reference ranges had ID given the predilection to ID in WB horses.^6^^,^^24–26^^,^^31^^,^^36^^,^^50^ Given that the significant differences between the ID and non-ID groups are consistent with LV remodeling consistent with mild hypertrophy, this should be considered in future studies.

Other variables, including abdominal fat, retroperitoneal fat, BCS, and adipokines, did not differ between the 2 groups (Table 4). CNS did vary significantly between the 2 groups with greater scores in horses with ID. This supports previous studies identifying CNS as a more sensitive indicator of ID than BCS.^28^ Another objective in this study was to investigate NIBP. Most horses of either the ID and non-ID groups were normotensive. Two horses, 1 from each group, were hypertensive based on the available literature in horses.^2^^,^^8^^,^^32^^,^^33^ Based on the low number of hypertensive horses in the study, no correlation could be established between NIBP, cardiac remodeling, and possible hypertrophy, and a single incidence of hypertensive cardiomyopathy was identified in the ID group.

The horses included in this investigative study were mildly affected with ID with only 2 horses having laminitis at the time of presentation. In the absence of laminitis the echocardiographic changes between mildly affected ID and non-ID horses can be inferred to be due to the presence of ID or another physiological process. Horses that that are more severely affected by laminitis or ID could be expected to have more prominent cardiac changes secondary to primarily ID, laminitis, pain and secondary hypertension or a combination of factors. The results of this study provide possible insight into the early pathophysiology of ID and the potential effects on the heart.

The intent of inclusion criteria of this study was to avoid inclusion bias. Horses with a previous diagnosis of ID or laminitis were excluded, thus preventing confounding factors that would influence the echocardiographic measurements. Only having 2 laminitic horses with normal NIBP measurements presents as a positive for the study as the investigators were able to evaluate cardiac variables independent of laminitic changes, ruling out the presence of pain related hypertensive cardiomyopathy.^8^ Both horses with laminitis were both within the ID group, with mild radiographic changes (*≤*5.1° rotation). It cannot be ruled out that the presence of laminitis in these 2 cases is not an incidental finding secondary to an underlying cause aside from ID, with lasting radiographic changes. This small number also acted as a limitation as the results of this study cannot be directly applied to horses with both ID and laminitis, and the study was unable to evaluate one of the initial objectives. Due to the study design, the results might not be able to be extrapolated to horses with more severe ID, and the echocardiographic changes might be more diffuse or severe in those instances.

This study provides evidence that cardiovascular changes might be considered to be included in the overarching definition of ID and EMS, and that LV remodeling and possible hypertrophy is associated with mild ID. The results of this study support an association between ID, LV remodeling, and possible cardiac hypertrophy.^23^ This study suggests that echocardiography is warranted in horses with ID with either elevated heart rates or changes in performance that cannot be linked to laminitis, although further investigation is needed to determine the effects of ID on the heart. Additionally metabolic diseases such as ID should be considered in future studies investigating cardiac variables in horses.

## Off-label antimicrobial declaration

Authors declare no off-label use of antimicrobials.

## Institutional animal care and use committee or other approval declaration

Approved by University of Pennsylvania Institutional Animal Care and Use Committee, NBC POAP number 807119.

## Appendix

### Animals

Informed owner consent was obtained for inclusion in the study. Exclusion criteria included use of a thyroid supplement, abnormal cardiac auscultation, and a previous diagnosis of ID, cardiac disease, or renal disease. Exclusion of horses with laminitis was based solely on history. Horses without a history of laminitis which were diagnosed with laminitis at the time of study commencement were included in order to investigate objective 3.

All portions of data collection were obtained by the same personnel.

A 2 means *t-*test power analysis was performed with a minimum of 6 horses per group required for adequate power when assessing for both change in blood pressure measurements (10 mmHg) and echocardiographic variables between groups (differences vary based on echocardiographic variables and were based on D’Fonseca et al.).^12^ Alpha was set to 0.05 with a power of 90%.

### History

History was recorded using a standardized questionnaire for all owners. This included recording of current medications, known previously diagnosed medical conditions (ie, cardiac murmurs or arrhythmias, history or previous diagnosis of laminitis, renal disease, insulin resistance, or pars pituitary intermedia dysfunction), current level of activity, and presenting complaint to the hospital. If an owner responded “yes” to a known diagnosis of a medical condition the horses were excluded from study inclusion.

### Physical variables

Each horse had a complete physical examination including comprehensive cardiac auscultation, body weight recorded in kg, body condition scoring (BCS, 0-9), and cresty neck scoring (CNS, 0-5) performed.^26^^,^^28^

### Oral sugar test and insulin concentration

An OST was elected to identify horses with ID given the dynamic nature of the test and ability to identify both hyperinsulinemia and insulin resistance components of ID. Horses were allowed free choice access to grass hay were withheld from grain or concentrates for at least 6 hours before the OST. Serum insulin concentrations were measured using a chemiluminescent assay (Siemens Immulite 2000) previously validated for use in horses.^51^ This laboratory recommended, assay-specific cut-off used in the current study for 60 and 90 min post OST (≤30 μIU/mL) is equivalent (based on assay comparison studies^51^^,^^52^) to the cut-off of > 45 μIU/mL that is recommended for the radioimmunoassay,^53^ which is based on work by Restifo and Frank.^54^^,^^55^ Classification of ID was based on the guidelines suggested by Restifo and colleagues (adjusted for measurement using the chemiluminescent assay and forming the basis of the (masked for reviewer) clinical laboratory references ranges): horses were considered ID positive if serum insulin concentration was ≥30 μIU/mL at 60 min (T60) or 90 (T90) min after oral administration of 0.15 mL/kg Karo Lite syrup.^54^ Horses were divided into ID and non-ID groups based on the results of the OST.

### Metabolic variables

Measurement of serum leptin, total, and high molecular weight (HWM) adiponectin concentrations were performed at T0 to assess for possible adipokine dysregulation. Leptin concentrations were attained using radioimmunoassay (Cornell diagnostic laboratory). Total adiponectin was measured using immunoturbidometric assay validated in the authors’ laboratory^56^ and similar to that previously validated in ponies^57^ and HMW adiponectin measured using ELISA,^58^ both previously validated for use in horses.

### Non-invasive oscillometric blood pressure measurements

Non-invasive oscillometric blood pressure monitoring (NIBP) (LifePak 12, Stryker, Portage, MI) was obtained from resting, unsedated horses based on previously established methods using a tail cuff and 5 sequential measurements.^32^^,^^34^ The measurements with the highest and lowest MAPs were excluded and the mean of the remaining 3 measurements utilized. Hypertension was defined as systolic pressure >150 mmHg and diastolic pressure >100 mmHg.^32^^,^^34^ Cuff width to tail circumference ratios of 0.4-0.6 was targeted. Blood pressure measurements were corrected using number of centimeters above the point of the shoulder and a constant coefficient of 0.77 mmHg.

### Radiographic imaging

Lateromedial foot radiographs were obtained of both front feet (Vet Rocket CX1, Santa Clara CA; Canon 801C detector, Irvine CA) with measurements taken along the lamellar lucent zone^59^ and founder distance measured. Measurements taken included the lamellar lucent zone at 3 locations: 5 mm distal to the extensor process, midway between the extensor process and solar margin, and 5 mm from the solar margin of the third phalanx, and dorsal hoof wall angle between the dorsal aspect of the third phalanx and the edge of the lucent zone (inner hoof wall) angle. Founder distance was measured from the proximal aspect of the extensor process to the coronary band. Measurements of > 8 mm of the 3 lucent zone measurements or > 3-degree dorsal hoof wall angle were considered consistent with a radiographic diagnosis of laminitis. Radiographs were reviewed by a large animal internist with specialization in laminitis.

### Retroperitoneal fat measurements

Fat measurements were obtained by transabdominal ultrasound (i8CX1 transducer and Toshiba Aplio i700 ultrasound system, Canon Medical Systems, Tochigi, Japan) in 2 locations; directly off midline in the cranioventral abdomen immediately caudal to the sternum and perirenal (around the right kidney).^60^^,^^61^ Measurements were obtained in triplicate. Perirenal fat measurements were reported as the mean with cranioventral abdominal fat measurements were reported as the greatest value obtained in 3 measurements.

### Echocardiographic measurements

Cardiac assessments were made using a standard cardiovascular platform (Vivid E95, GE Healthcare, Horten, Norway) and a M5S sector transducer (GE Healthcare), with simultaneous single-lead ECG recording. Fractional shortening (FS), mean wall thickness (MWT = LVFWd+IVSd), left ventricular mass (LVM = 1.04^*^ (((LVIDd+LVFWd+ IVSd)^3^-(LVIDd^3^))-13.6), and relative left ventricular wall thickness ((LVFWd+IVSd)/LVIDd) were calculated based on the previously reported formulas.^29^^,^^30^ Pulsed wave tissue Doppler imaging (PW-TDI) was used to obtain LV myocardial velocities and cardiac cycle intervals using the right parasternal short axis view of the LV at the level of the chordae tendineae as previously reported.^31^ The sample volume was placed on the left ventricular free wall. Diastolic function was assessed based on measurements of IVRTm (isovolumic relaxation time), Em (early-diastolic LV wall motion velocity during the phase of rapid ventricular filling), and Am (late-diastolic LV wall motion velocity at the time of atrial contraction). Em/Am was calculated.

For Pulsed wave tissue Doppler imaging a sample volume of 6.0 mm width at a frequency of 2.7 MHz was used.

The LV internal dimension measurements were corrected for body weight using allometric scaling as previously described per diameter = measurement/body weight(kg)^1/3*^500^1/3^.^36^

Echocardiographic images were reviewed by a large animal veterinarian with advanced cardiology training.

### 2D speckle tracking

A total of 7 ID horses and 7 non-ID horses underwent additional analysis including 2DST. 2D speckle tracking (2DST) of the LV wall was performed in all horses as previously described^49^ using right parasternal short-axis images of the LV at the level of the chordae tendineae.

Analysis was performed using the Q-analysis feature of EchoPAC software (GE Healthcare). For each horse, 3 non-consecutive cardiac cycles were analyzed and averaged. End-diastole was defined as onset of the QRS complex on ECG. The region of interest (ROI) was set by tracing the endocardial border at end-systole, and then adapting the width of the software-generated ROI. The software divided the myocardium into 6 segments analysis based on human heart orientation (AntSept, Ant, Lat, Post, Inf, and Sept). In horses, Ant and AntSept correspond to the interventricular septum, Lat and Post to the cranial left ventricular free wall, and Sept and Inf to the caudal left ventricular free wall. Tracking quality was calculated by the software and visually verified by the operator, with adaptations if necessary, until satisfactory tracking was achieved. Radial displacement at end systole was recorded in addition to peak systolic radial strain associated with each segment. Echocardiographic images were reviewed by a large animal veterinarian with advanced cardiology training.

### Statistical analysis

All continuous data were assessed for normality using Shapiro–Wilk statistical analysis with the data being non-normally distributed. Descriptive analyses included computation of means, medians, ranges of continuous variables, and tabulation of categorical variables. For each outcome, the marginal means analysis involved a single, pre-specified contrast between 2 groups. Consequently, only 1 pairwise comparison was performed per model, and no adjustment for multiple comparisons was applied, as multiplicity corrections are intended to control the family-wise error rate when multiple simultaneous contrasts are evaluated. Allometrically scaled echocardiographic variables were analyzed using the same modeling approach as the unscaled measurements.

**Table 1A:** Lateromedial radiographic measurements.

Age	Sex	Group	lucent zone 1 (mm)	lucent zone 2 (mm)	lucent zone 3 (mm)	Dorsal hoof wall angle (°)	Laminitis criteria met?
**7**	MC	Non-ID	7.7/8.1	6.8/7.6	7/8.2	0/0	N
**6**	F	Non-ID	6.7/6.5	5.7/5.7	6.6/6.5	0/0	N
**11**	F	Non-ID	5.5/5.8	6.4/6.3	5.8/6.5	0/1.5	N
**5**	F	Non-ID	8.1/8.2	7.8/7.4	8.3/7.7	1/0	N
**6**	MC	Non-ID	6.3/6.9	6.1/6.2	7.4/6.3	1/0	N
**6**	F	Non-ID	6.9/6.8	6.6/6.5	6.6/6.8	0/0	N
**9**	MC	Non-ID	6.7/7	6.9/6.8	7.9/7	1/0	N
**5**	F	Non-ID	7.3/7.3	7.2/7.3	7.9/7.9	1.8/1.5	N
**5**	F	Non-ID	7.3/7.7	7.6/7.5	8.7/8.3	1.2/1.5	N
**11**	MC	Non-ID	5.8/6.3	5.8/6.2	5.9/6.4	0/1	N
**8**	F	Non-ID	6.9/6.8	6.4/6.6	7.6/7.7	1/1.2	N
**12**	MC	ID	6.4/6.6	6.9/6.1	6.1/5.9	4/0	**Y**
**9**	MC	ID	6.6/7.4	5.9/6.8	7.3/7.3	1/1	N
**9**	F	ID	6.6/7	5.7/6.1	5.7/5.8	0/0	N
**11**	MC	ID	7.3/6.4	7.5/6.1	7.9/6.7	0/0	N
**15**	F	ID	8.4/7.4	7.6/7.3	7.6/8.1	0/1	N
**10**	MC	ID	6.9/6.7	6.5/5.9	7.7/6.2	2/0	N
**6**	F	ID	6.8/7.2	6.5/7	7.5/6.5	0/0	N
**13**	F	ID	6.7/7.4	7.3/8.3	8.5/9.6	3.8/5.1	**Y**

Measurements for each horse included in the study. Age and sex are included in the first and second columns. Measurements are recorded as left front/right front for the lucent zone measurements, dorsal hoof wall angle, and whether laminitic criteria have been met as yes (Y) or no (N). (MC, male castrated; F, female; ID, insulin dysregulate).

## Abbreviations

bpm: beats per minute
BCS: body condition score
cTnI: cardiac troponin I
CNS: cresty neck score
Em: early-diastolic LV wall motion velocity during the phase of rapid ventricular filling
EMS: equine metabolic syndrome
FS: fractional shortening
HR: heart rate
HMW: high molecular weight
ID: insulin dysregulation
IVSd: interventricular septum at end diastole
IVSs: interventricular septum at end systole
IVRTm: isovolumic relaxation time
Am: late-diastolic LV wall motion velocity at the time of atrial contraction
LV: left ventricle
LVFW: left ventricular free wall thickness
LVFWd: left ventricular free wall thickness at end diastole
LVIDd: left ventricular internal diameter at end diastole
LVM: left ventricular mass
MAP: mean arterial pressure
MWT: mean wall thickness
NIBP: non-invasive oscillometric blood pressure
OST: oral sugar test
PW-TDI: pulsed wave tissue Doppler imaging
RWT: relative wall thickness
TDI: tissue Doppler imaging
WB: Warmblood
2DST: 2D speckle tracking
